# Hospital-initiated community pharmacist medication review post-discharge

**DOI:** 10.1007/s40520-026-03416-1

**Published:** 2026-06-11

**Authors:** Louise Clarkson, Bernadette Pollard, Shu-Kay Ng, Eve Finn, Alfred K Lam, Tien K Khoo

**Affiliations:** 1 School of Medicine & Dentistry, Griffith University, 4222 Gold Coast, QLD Australia; 2 Northern New South Wales Local Health District, NSW Lismore, Australia; 3 Graduate School of Medicine, University of Wollongong, NSW Wollongong, Australia

## Abstract

**Background:**

Drug related problems (DRPs) are commonly encountered by elderly patients on discharge from hospital. Further research is needed into interventions for risk reduction.

**Aims:**

To investigate DRPs post-discharge and assess the Australian hospital-initiated community pharmacist medication review (HIMR) service for benefit.

**Methods:**

Retrospective analysis of the Ballina Hospital HIMR service from 20/02/2023 to 20/02/2024 was completed. Data included patients baseline demographics, discharge medications, time to HIMR, DRPs identified, actions taken, and pharmacists’ recommendations for medication changes.

**Results:**

The study cohort (*n* = 143) had a mean age of 82.2 ± 10.9 years and were prescribed an average of 9.4 ± 4.0 regular medications with 6.0 ± 4.6 medication changes at discharge. The initial HIMR (HIMR1) was completed 7.6 ± 6.1 days (median: 6.0) post-discharge, identifying non-adherence in 43% and possible adverse drug reactions in 22% of patients. Eleven patients (7.7%) received pharmacist interventions of moderate/high significance. Eighty-three follow-up visits (HIMR2) were attended. The HIMR2 reports showed 18.8% of medication change recommendations suggested by the pharmacist at HIMR1 were implemented.

**Discussion:**

Significant interventions at HIMR1 were more likely to be experienced by patients ≥ 80 years, when comorbidity increased and when patients were reviewed sooner. Although multiple DRPs were identified, medication change suggestions made at HIMR1 were infrequently actioned in primary care.

**Conclusions:**

DRPs and non-adherence were common in the population reviewed. HIMR was valuable for resolving immediate DRPs and assisting medication management, especially when performed nearest discharge. Further optimisation of this service to strengthen engagement with primary care practitioners is recommended.

**Supplementary Information:**

The online version contains supplementary material available at 10.1007/s40520-026-03416-1.

## Introduction

There is a significant risk of medication-related harm during the transition of care to the community following hospital discharge [1, 2]. Contributing factors may include discrepancies in medication lists, inappropriate prescribing, and inadequate patient education, which can result in treatment errors [3]. Older adults are particularly vulnerable because of the increased prevalence of comorbidities and hospital admissions, along with physical and cognitive changes related to ageing [4]. The risk of medication-related harm is considered to be highest within the first 10 days following discharge from hospital [5]. Hospital-discharge may also inadvertently contribute to inappropriate polypharmacy; Discharge reconciliations may include unintended medications and in some cases patients can be continued on medications started in hospital for an acute illness despite no indication for chronic use [6–8].

A medication review conducted by a pharmacist after hospital discharge may facilitate a safer transition from hospital to home by providing the patient with education and support regarding medication use, as well as resolving drug related problems (DRPs). International studies examining the effectiveness of pharmacist-led medication reviews and transition of care interventions have reported benefits, including the resolution of medication discrepancies and reduction in hospital readmissions in some studies [6, 9, 10]. In Australia, there have been several approaches to post-discharge medication reviews. For example, there is the Monash Health Hospital Outreach Medication Review (HOMR) initiative (which involves a home visit post-discharge from a hospital-employed pharmacist) which has found a 25% reduction in hospital re-admissions among older patients [11]. However, the access to hospital-funded transitional care pharmacists in Australia is very limited, especially in smaller hospitals. Post-discharge pharmacist reviews coordinated by a pharmacist working within a general practitioner (GP) surgery have also reduced the incidence of hospital re-admissions over a 12-month period [12]. Other hospitals have utilised hospital-based, post-discharge clinics with some perceived benefit [13].

Since 2020 the Australian Commonwealth have funded a hospital-initiated medication review (HIMR) program which utilises community-based pharmacists. Under this program, a hospital-based medical specialist can refer a patient on discharge from hospital to a community-based pharmacist credentialled in medication reviews for a home visit. Protocols for the HIMR pathway are available from Advanced Pharmacy Australia [14]. After completion of a home-based medication review with the patient, the pharmacist prepares and distributes a report. This report is then discussed between the patient and their GP (or patient and medical specialist if face-to-face follow-up was planned post discharge). As per the program rules the GP or medical specialist should then work with the patient to complete a written medication management plan.

HIMRs are relatively new, infrequently utilised and are the subject of investigation [15, 16]. The HIMR pathway was integrated into the discharge process at Ballina Hospital in 2023 for patients deemed to be high-risk of drug related problems. The primary objective of this study was to review the scope of DRP’s experienced by our perceived ‘high-risk’ patients on discharge and examine if a HIMR provided benefit. All patients who received a HIMR in the first year of implementation were examined. Primary areas for review included (a) DRPs identified by the pharmacist at the HIMR and (b) actions taken/interventions made by the visiting HIMR pharmacist and any immediate DRP’s resolved. Other primary areas for review included whether the HIMR report was effective for influencing medication changes (and particularly deprescribing) in primary care with the patients GP post-discharge. Secondary areas for review included characteristics of the patients receiving the most benefit from HIMR and consideration of potential improvements to the HIMR pathway.

## Methods

### HIMR service

Ballina Hospital implemented a pilot HIMR service on 20/02/23 in accordance with the Advanced Pharmacy Australia (AdPha) (formerly the Society of Hospital Pharmacy Australia (SHPA)) guidelines (hospital-initiated_medication_reviews_hppu.pdf (shpa.org.au) [14]. At the time of referral, the treating medical specialist confirmed that each patient met all of the following criteria: (a) the patient is at risk of medication misadventure in the immediate post-discharge period, (b) the patient would benefit from review with a pharmacist in the immediate post-discharge period and (c) the normal pharmacist discharge activities are insufficient to meet the patient’s needs and would be complimented by home medication review.

They also had to confirm that the patient met two of the following indicators for referral: (a) the patient is cognitively impaired and manages their own medicines, (b) the hospital pharmacist assessment reveals risk with intentional OR unintentional non-adherence, (c) the patient has been initiated on medication with a narrow therapeutic index during admission, (d) the patient has had recurrent admissions to hospital (e.g. 2 within 6 months), (e) there have been changes to regular drug regimen made during admission by hospital doctor with potential for confusion (excluding short-term courses under 14 days), (f) the patient takes more than 12 doses of medicines per day, (g) the patient is on 5 or more regular medicines, (h) the patient is from a culturally or linguistically diverse background, or (i) the patient has a chronic and/or complex disease. Suitable patients that would meet the criteria for a HIMR could be identified by any member of the hospital health care team (including the ward pharmacist, nurses, discharge planners and doctors). The HIMR referral had to be completed by the patients treating medical specialist as per program rules. No restriction was given on age of the patient. However, since the patients attending Ballina Hospital are usually of older age, we were aware that this would likely be our study population. The referring physician could also request a further follow-up visit with a pharmacist one month after the first visit as per HIMR program rules [14].

The HIMR referrals were directed to the home medication review accredited pharmacist nominated by the patient’s regular community pharmacy. The accredited pharmacist was encouraged to communicate with the patient/caretaker to arrange a time for the first HIMR as soon as possible, ideally within 5 days post-discharge. The HIMR was then completed in the patient’s home and a report prepared.

Under the existing service model, copies of the HIMR reports were provided to the patient’s GP, community pharmacy, and referring medical specialist. In our pathway, the patient’s usual GP was the person best placed to review the HIMR report. The medical team were encouraged to provide adequate information in the discharge handover to enable the GP to complete a medication management plan with their patient at their next face-to-face appointment after the HIMR service.

### Review of the HIMR service

All patients referred for an HIMR upon discharge from Ballina Hospital over the 12-month period from 20/02/2023 to 20/02/2024 were reviewed. This study was approved by the Northern New South Wales Human Research Ethics Committee (reference: QA454).

The time taken until completing the service (and follow-up service, if applicable) was documented. Core baseline medical data (i.e., demographic information, comorbidities, and discharge medications) was collected from the electronic medical records. Morbidity was assessed using the Charlson Comorbidity Index (CCI) [17]. High-risk medications were recorded, including those classified as high risk by the Australian Commission on Safety and Quality in Healthcare (i.e., insulin, opioid analgesics, anti-coagulants, or antipsychotic medicines) as well as medications potentially inappropriate for use in older adults (i.e., anticholinergics, hypnotics, and non-steroidal anti-inflammatory drugs) [18, 19]. Strong anti-cholinergic medication was defined as those with an anti-cholinergic burden score of ≥ 3 [18].

All reports (including initial and follow-up reports) received back from the community accredited pharmacists for the HIMRs completed were independently retrospectively studied by two experienced hospital pharmacists. Both pharmacists had over 20 years of pharmacy practice and previous expertise in completing medication reviews in community and hospital sectors. There was a high degree of agreement between the pharmacists with the categorisation of DRPs identified, medication changes recommended to the GP and actions/interventions completed by the pharmacist at the initial HIMR (HIMR1). There was also a high degree of agreement with review of the HIMR2 follow-up reports; These were examined by both pharmacists to see if any medication change recommendations from HIMR1 had been implemented by the patients GP. If an identical patient was referred for a second time (i.e., received the initial service twice) during the 12-month study period, which was the case for two patients, then the reports from the second service were removed from the analysis.

The DRPs identified, and medication changes recommended by the pharmacist in the HIMR reports were recorded and categorised using a modified DOCUMENT system, a validated tool used to report DRP and clinical interventions [20]. Modifications to the DOCUMENT DRP classification system were previously used in other studies that examined HMR reports [21]. DRPs were classified into the following categories: drug selection, overdose or under-dose, adherence, suboptimal response to medication or medication change, unclassifiable, and toxicity or adverse drug reaction. In-line with current taxonomy, adherence was further classified into implementation of dose regimen, and discontinuation of prescribed therapy (noting all medications had been first initiated while in hospital) [22]. A further adherence classification was added for those patients that restarted home medications that had been ceased during hospital admission. Education was not included as a DRP category since this information could not be accurately extracted from the HIMR reports.

Actions/interventions completed by the pharmacist during the HIMR were also classified by the DOCUMENT system, based on clinical significance: mild (i.e., consequences to the patient are that they have improved adherence or the intervention improved/prevented a minor symptom), moderate (i.e., if the intervention did not occur, the patient would likely need a medical consult because of the consequences), and high (i.e., if the intervention did not occur, the patient would likely need a hospital visit because of the consequences) [20].

### Statistical analysis

Data are presented as frequency (percentage) for binary or categorical variables and mean ± standard deviation (SD) for continuous variables. Continuous variables were assessed for normality using the Kolmogorov–Smirnov and Shapiro–Wilk tests. Participant demographics and medication-related characteristics were evaluated along with the presence of DRPs and significant pharmacist interventions. The chi-squared test was used for between-group comparison of categorical variables, whereas the independent-samples *t*-test was used for between-group comparison of continuous variables. The Mann–Whitney *U* test was used to evaluate medication-related characteristics, such as the number of days before the HIMR service, in which the data were not normally distributed. Statistical analysis was performed using IBM SPSS Statistics Version 30 (IBM Corp., Armonk, NY, USA).

## Results

During the 12-month study period, 169 patients were referred for a HIMR with an accredited community pharmacist from 5 physicians, among whom 2 patients were referred twice. HIMR reports were received back from 5 community accredited pharmacists, and a total of 143 patients received 145 services. Reports from the initial HIMR visits (HIMR1) show they were completed at a mean of 7.6 ± 6.1 days (median: 6.0; range: 0–38) post-discharge. Reports were received for 83 follow-up HIMR visits (HIMR2) which were conducted after a mean of 56.5 ± 17.1 days (median: 53; range: 34–128) post-discharge. For twenty-six referrals, no HIMR report was received back so they could not be included in the analysis.

The participants reviewed had multiple morbidities (mean CCI: 4.6 ± 2.9), were older individuals (mean age: 82.2 ± 10.9 years), and predominantly female (57.3%) (Table 1). Upon discharge, these patients had a high degree of polypharmacy (mean number of regular medications: 9.4 ± 4.0), with many taking high-risk medications. Additionally, these patients had a mean number of 6.0 ± 4.6 changes to their medication, including medications that were started, ceased, or changed in dose.

**Table 1:** Characteristics of the study cohort at the time of discharge

Item	Patient Demographics (*n* = 143) Mean ± SD (range) or frequency (%)
Age at discharge, years	82.2 ± 10.9 (48.7–103.0)
Gender	
*Female*	82 (57.3%)
*Male*	61 (42.7%)
Charlson Comorbidity Index (CCI)	4.6 ± 2.9 (0–14)
Total number of medications at discharge (*n* = 142)*	11.9 ± 4.5 (3–25)
Number of regular medications at discharge (*n* = 142)	9.4 ± 4.0 (2–25)
Number of patients prescribed at least 10 regular medications	67 (47.2%)
Total number of changes to home medications on discharge (including medications that were started, ceased, or changed) (*n* = 142)	6.0 ± 4.6 (0–25)
Using a dose administration aid packed by the pharmacy	80 (55.9%)
High-risk medications prescribed upon discharge (regular or as required)	
Insulin	15 (10.6%)
Anti-coagulants	61 (43.0%)
Strong anti-cholinergic	15 (10.6%)
Benzodiazepines	17 (12.0%)
Opioids	32 (22.5%)
Non-steroidal anti-inflammatory drugs	4 (2.8%)
Anti-psychotics	2 (1.4%)

^*One patient did not have either a pharmacist list or a discharge medication summary list on discharge.^

Among the 143 HIMR1 reports, reduced adherence was identified as a potential problem in 62 patients (43.4%) (Table 2). Based on the pharmacists’ evaluation, 31 patients (21.7%) were taking unnecessary or inappropriate medication, while 12 patients (8.4%) were prescribed a dose that was either too high or too low. Additionally, 14 patients (9.8%) were thought to have had a suboptimal response to medication (or medication change), and 31 patients (21.7%) needed further evaluation for possible adverse drug reactions.

**Table 2:** Types of drug-related problems highlighted in the HIMR1 report

Type of Drug-Related Problem	Number of HIMR1 reports (*n* = 143) frequency (%)
Drug Selection (*unnecessary or inappropriate medication identified*)	31 (21.7%)
Over- or Under-dose (*problems related to the prescribed dose of a medication*)	12 (8.4%)
Adherence *(not taking medications as prescribed)*	62 (43.4%)
*Discontinuation of prescribed therapy*	37 (25.9%)
*Incorrect implementation of dose regimen*	34 (23.8%)
*Self-restarting medication that was ceased in hospital*	7 (4.9%)
Undertreated *(suboptimal response to medication/medication change)*	14 (9.8%)
Non-classifiable *(documentation errors*,* new medical conditions for review*,* etc.)*	50 (35.0%)
Toxicity or possible adverse drug reactions	31 (21.7%)

In 95 reports (66.4%), the pharmacist educated the patient or intervened to improve medication management. These actions are described using the modified DOCUMENT system in Table 3. Eleven patients (7.7%) received actions or interventions deemed to have moderate or high significance by the two independent reviewers.

**Table 3:** Actions described in the initial hospital-initiated medication review (HIMR1) report

Clinical significance of actions implemented during HIMR1	Number of HIMR reports (*n* = 143) *n* (%)	Examples of documented actions
Mild	90 (63.0%)	• *Medicine organisation/clean up* • *Inhaler technique corrected* • *Aids such as spacers or pilbobs organised* • *Filled dosette box* • *Organised provision of the glucometer* • *Requested visual aid stickers on the dose administration aid (DAA)* • *Provided fluid management/daily weights guide* • *Blood pressure checks* • *Change in DAA style organised with the pharmacy* • *Assisted with change of pharmacy* • *Corresponded with care providers* • *Contacted the dietician* • *Contacted pulmonary rehabilitation for an appointment* • *Helped with the application for continence aids* • *Collected and delivered antibiotics* • *Provided oral and printed education on hypoglycaemia management* • *Information provided on administering medications for Parkinson’s disease*
Moderate	6 (4.2%)	• *Corrected accidental double dose of metoprolol taken by the patient* • *Corrected accidental double dose of paracetamol and omission of furosemide (patient assumed it was in DAA)* • *Informed pharmacy of error with apixaban being packed once daily* • *Identified and corrected the subtherapeutic dosing of apixaban* • *Corrected the DAA so that the patient took their antibiotics following hospital admission for a urinary tract infection* • *Corrected the incorrect dose of bisoprolol*,* which had been increased at the hospital without the patient’s knowledge*
High	5 (3.5%)	• *Removed ceased anti-hypertensive medication from patients with cognitive impairment and a high risk of falls* • Removed temazepam (*N* = 75 tablets) from a frail older patient with a history of overuse; sent to the pharmacy for inclusion in the DAA • *Replaced a Webster pack containing ceased medication*,* of which the patient was unaware*,* with a new DAA* • *No salbutamol was supplied at discharge*,* leaving an older*,* confused patient short of breath; the pharmacist helped organise a replacement* • *Coordinated the start of the DAA with the pharmacy for a vulnerable patient with poor adherence and at high immediate risk of misadventure*

^Mild: consequences to the patient including improved adherence or the intervention improved/prevented a minor symptom.^

^Moderate: if the intervention did not occur, the patient would likely require a medical consult because of the consequences.^

^High: if the intervention did not occur, the patient would likely require hospitalisation because of the consequences.^

The 143 HIMR1 reports contained 225 recommendations for medication change (Table 4). At least one recommendation was present in 112 (78.3%) of the reports. Reducing the dose of a medication was the most frequent recommendation (31.1%) and was present in 40.6% of the reports. A second follow-up pharmacist visit (HIMR2) was conducted for 82 patients. Analysis of the 82 HIMR2 reports showed that 25 of the 133 (18.8%) medication change recommendations made to the GP by the pharmacist for these patients in their HIMR1 reports were actioned. The recommendation for increasing the dose of a medication was actioned for 4 of 8 (50.0%) recommendations. Ceasing or reducing the dose of a medication was actioned for 4 of 18 (22.2%) or 9 of 46 (19.6%) recommendations respectively.

**Table 4:** Recommended medication changes to the GP in the HIMR1 reports

Medication change recommendation	Medication change recommendations in all HIMR1 reports (*n* = 143)	Medication change recommendations in HIMR1 reports for patients receiving HIMR1 and HIMR2 (*n* = 82)*	Number of recommendations made in HIMR1 attended in HIMR2 (Frequency %)
Total number of recommendations	225	133	25 (18.8%)
Increase in medication dose	14 (6.2%)	8 (6.0%)	4 (50.0%)
Decrease in the medication dose	70 (31.1%)	46 (34.6%)	9 (19.6%)
Cessation of medication	28 (12.4%)	18 (13.5%)	4 (22.2%)
Initiate/restart medication	33 (14.7%)	15 (11.3%)	2 (13.3%)
Switch to an alternate medication	36 (16.0%)	23 (17.3%)	2 (8.7%)
Change in the drug formulation/schedule	44 (19.6%)	23 (17.3%)	4 (17.4%)

^*There were 83 HIMR2 reports received; however, the second report was excluded from the analysis for patients who received the service twice.^

Table 5 describes the medication characteristics of 80 participants at HIMR2 (2 participants were excluded from this analysis due to incomplete medication lists). At HIMR2 an extra 3 participants had been initiated on a dose administration aid packed by the pharmacy and tablet burden had reduced by an average of 2 tablets. The number of medications remained consistent. The number of people using a benzodiazepine (either regular or when required) had increased by 6 participants.

**Table 5:** Medication Characteristics of the patients who received HIMR2

Medication Characteristics	Discharge Medications mean (SD, range) or frequency (%)	Medications at HIMR2 mean (SD, range) or frequency (%)
Total Number of Medications	12.0 (4.6, 4–25)	12.1 (5.1, 1–25)
Number of Regular Medications	9.2 (4.1, 2–25)	9.5 (4.1, 1–22)
Tablet Burden of regular medications	14.0 (7.3, 2–35)	12.2 (6.4, 1–27)
Using a dose administration aid packed by the pharmacy	45 (56.3%)	48 (60.0%)
High-risk medications prescribed on discharge (regular or as required)		
Insulin	8 (10.0%)	8 (10.0%)
Sulphonylureas	2 (2.5%)	3 (3.8%)
Anticoagulants	35(43.8%)	34 (42.5%)
Strong Anticholinergics	11 (13.8%)	11 (13.8%)
Benzodiazepines	**12 (15.0%)**	**18 (22.5%)**
Opioids	19 (23.8%)	20 (25%)
NSAIDS	2 (2.5%)	3 (3.8%)
Antipsychotics	0	0

^*n* = 80 for medication comparisons (82 HIMR2 services completed, however 2 reports did not have complete medication lists).^

Supplementary Tables 1 and 2 present between-group comparisons of patient characteristics, medication taken, and the time required for HIMR service, along with non-adherence, drug selection concerns, ADR concerns, and moderate or high intervention. Patients who received moderate or high intervention were ≥ 80 years and had increased comorbidities (mean CCI: 6.6) compared with those who received no or mild intervention (*p* = 0.019 and *p* = 0.016, respectively). In addition, a shorter time to HIMR service was associated with receiving an intervention of moderate/high importance (*p* = 0.049). An increase in the number of medications was associated with possible adverse drug reactions (*p* = 0.010), and patients taking medications with strong anti-cholinergic properties were consistently flagged for drug selection concerns (*p* = 0.002).

## Discussion

Studies have indicated that the initial 10-day period post-discharge is associated with the highest risk of medication-related problems [5, 23]. During the HIMR pilot at Ballina Hospital, 143 patients received a home visit from a community-accredited pharmacist after an average of 7.6 days (median: 6 days) following hospital inpatient discharge. Intervention within 10 days post-discharge was achieved in 76% of the participants. We found that for this study population, the sooner the HIMR was performed, the more likely an important intervention would be made (*p* = 0.049). We would suggest that targeting future interventions to the first couple of days post-discharge may be of most benefit.

It has been previously documented that the risk of post-discharge adverse drug events increases with a higher number of prescribed medications, older age, and multiple comorbidities [24]. Similarly, our results revealed that age ≥ 80 years (*p* = 0.019) and increasing comorbidity (*p* = 0.016) were associated with significant medication-related concerns at HIMR1 requiring immediate intervention from the pharmacist. These actions performed by the pharmacist in approximately 1 out of every 13 home visits were judged to have moderate/high importance in preventing adverse health consequences. Additionally, an increase in the number of patient medications (*p* = 0.010) was associated with possible adverse drug reactions identified by the pharmacist, as documented in 31 patients (22%). Some examples highlighted for GP review included postural dizziness with alpha-blocker use, drug-induced hyponatraemia, and constipation from oral iron supplementation. Pharmacists also flagged 14 patients (9.8%) believed to have a suboptimal response to medication (or medication change) during the immediate post-discharge period, such as persistent/worsening ankle oedema (despite initiation of a loop diuretic or after diuretic withdrawal) and an increased heart rate after cessation of beta-blocker therapy. These patients were advised to promptly seek medical advice.

This study found no association between the incidence of adverse drug reactions and medication class, highlighting that medication related harm can occur for medications other than just those deemed ‘high risk’. In accordance with the Beers Criteria for potentially inappropriate medication use in older adults, patients taking highly anti-cholinergic medications had the highest likelihood of having documented drug selection concerns by the pharmacist [18]. Unfortunately, at HIMR2, the same number of patients were still taking these medications.

Pharmacists identified that 43% of the cohort had suboptimal adherence with at least one medication. As described in Table 2, patient education and interventions were attempted to improve adherence. Reduced adherence was multifactorial within our comorbid geriatric study population, and it was not significantly more prevalent in patients taking more medication. Inhaled medications were frequently associated with reduced adherence, with 16 (34%) patients incorrectly using inhalers prescribed upon discharge. This finding is consistent with that of previous studies reporting adherence rates of only 20%–60% with inhaled medications in chronic obstructive pulmonary disease [25]. In response to adherence concerns, pharmacists frequently suggested changing the route or administration schedule of a medication to reduce complexity (25.9%), which included using combination products, alternate inhaler devices, and dose administration aids. At HIMR2, three additional patients had changed to using a dose administration aid packed by their pharmacy and although the average number of medications remained the same, the average tablet burden had reduced by 2 due to use of combination products.

The pharmacists suggested ceasing certain medications in 15% of patients, reducing the dose in 41%, and switching one drug to an alternative in 24%. Additionally, the pharmacists recommended restarting or initiating a new medication in 20% of patients, including medication for optimising medical therapy for chronic health conditions (e.g., heart failure, chronic obstructive pulmonary disease, and osteoporosis). One of the goals of this study was to determine whether pharmacists could promote medication changes in primary care through the HIMR. However, only 19% of the pharmacist’s recommendations were actioned at HIMR2, suggesting the HIMR1 reports had a limited impact on GP prescribing. Four patients did have their medication ceased (from 18 suggestions) and 9 patients had a dose decreased (from 46 suggestions). Although only a small number of suggestions were actioned, they may have provided significant benefit to the patients involved. Medications ceased included the alpha-blocker identified by the pharmacist as causing postural dizziness, resulting in symptom improvement. Proton-pump inhibitors were the most frequently reduced medications, potentially reducing the risk of adverse effects associated with long-term use [26]. The acceptance rates of pharmacist recommendations in the context of HMRs are widely inconsistent in literature, ranging from 27% to 93% [27]. The reasons for this are likely to be multifactorial and include instances where more generalised pharmacist recommendations are deemed inappropriate by the GP, who has complete knowledge of their patients’ medical history. The extent of collaboration between the pharmacist and GP may also influence acceptance. In the current model—in which the referral is hospital-initiated—the GP may be less engaged with the process. Notably, the pharmacist’s recommendations may also be non-interventional in nature and unable to be captured by this study. 68% of HIMR1 reports included additional suggestions for medication monitoring or health improvement.

The 80 patients with medication reviewed at HIMR1 and HIMR2 (mean 7.6 and 56.5 days post-discharge respectively) were still prescribed an average of 12 medications (9 being regular medications) at the second visit. Notably, the number of participants prescribed an opioid medication (nearly 2-months after their hospital admission) increased by one and the number of participants prescribed a benzodiazepine had increased by 6. Without full knowledge of these individuals’ past medical history and goals of care, we cannot assess the appropriateness of continued polypharmacy; it is possible that all medications were necessary for optimal care. However, we should also consider that deprescribing within primary care is known to be a difficult and complex process with barriers such as a lack of evidence-based guidelines, consultation constraints and patient fear of negative consequences [28, 29]. Professional collaboration between health professionals (such as a pharmacist and GP) has been previously identified as a deprescribing enabler, although as previously noted, we were unable to demonstrate this in our study [29].

During the study period there were also 26 referrals (15.4%) completed for patients that were not actioned by the accredited community pharmacist. There is no requirement within the current HIMR pathway for the accredited pharmacist to document or communicate reasons for non-service. These 26 patients had no formal documentation to explain why they did not receive a HIMR service and no further follow-up was instituted. A documentation gap in the HIMR pathway was identified as a significant area for improvement. Reasons for non-service that were fed back by the accredited pharmacists included being referred patients who were admitted into an aged are facility or readmitted back into the hospital system before the service could take place, patients that declined the service after discharge (despite consenting to the service at the time of referral) or being referred patients they were unable to contact. These 26 patients, who were referred as they were deemed to be at risk of medication related problems, could unfortunately not be captured by the HIMR intervention.

Considering the scope of medication-related risks after hospital discharge, pharmacist home visits, particularly when completed immediately after discharge, can be very valuable. Our findings suggest that patients ≥ 80 years and those with significant comorbidities may benefit the most. In 95 reports (66.4%), the pharmacist specified that they educated the patient or intervened to improve medication management. Given the nature of the HIMR intervention (a conversation at home) and the formal structure of a HIMR report for the GP, it is likely that additional education was also provided and not documented (generally only the most important issues are highlighted to the GP to avoid a lengthy wordy report). For this reason, the full educational value of HIMR and benefit to the patient is likely to have been undervalued. We were able to clearly capture that the pharmacists addressed some significant medication concerns and errors during the post-discharge period that may have prevented adverse health outcomes, in addition to highlighting multiple medications for review by the GP. However, a HIMR completed by an accredited community pharmacist through this pathway has notable limitations, such as the low acceptance rate of pharmacist recommendations by the patients GP. To expand the benefit of the HIMR service, further research should explore ways of engaging primary care practitioners. When the pharmacist completing the medication review is integrated within a GP clinic, the acceptance of medication change recommendations can be increased [9, 24]. A pharmacist engaged with a GP clinic may also be better placed to access and encourage the difficult to reach patients Further studies could also explore the benefits of standardised HIMR reporting or introducing an individualised comorbidity-focused HIMR process. This approach has already shown potential value for patients with chronic heart failure [25].

### Limitations

Twenty-six patients were referred for a HIMR and no report received. These patients could not be included in the data analysis. Data extraction was limited by the heterogeneous nature of the HIMR reports, and some recommendations were open to interpretation. To minimise bias, each report was reviewed and categorised separately by two independent pharmacists. Many education-related pharmacist interventions were unable to be captured from this study, which may have undervalued the benefit of HIMR. Moreover, not all patients underwent a second HIMR (HIMR2). During HIMR2, medication changes were assumed to have been made in response to the pharmacist’s suggestion, but evidence to support this claim is lacking. Lastly, no economic evaluation was done, and the impact of interventions on patient outcomes was not assessed.

## Conclusions

There is a need for intervention to reduce medication-related harm to elderly patients at the time of discharge, with intervention performed closest to discharge being of most benefit. A significant degree of medication non-adherence was observed in this select ‘high-risk’ patient cohort referred for HIMR and multiple drug-related problems identified. The HIMR service was beneficial for resolving immediate medication-related issues and errors and assisting patients with medication management. However, the HIMR service in its current format was unable to reach all patients and was largely ineffective at influencing medication changes or promoting deprescribing in primary care. Further research and optimisation of this service to strengthen engagement with primary care practitioners is recommended.

## Electronic Supplementary Material

Below is the link to the electronic supplementary material.

Supplementary Material 1

## Data Availability

Data available on request due to privacy/ethical restrictions: The data that support the findings of this study are available on request from the corresponding author, LC, subject to ethical approval. The data are not publicly available due to their containing information that could compromise the privacy of the research participants.
