# Supplementary material for: Hospital-initiated community pharmacist medication review post-discharge
Source: Aging Clin Exp Res. 2026 Jun 11;38(1):179. doi: 10.1007/s40520-026-03416-1 (PMC13503446; doi:10.1007/s40520-026-03416-1)
Supplement: Supplementary file 1 [file 40520_2026_3416_MOESM1_ESM.docx]

**Supplementary Table 1. Patient factors and pharmacist-identified drug-related problems at HIMR1**

|  | No adherence concerns  (n = 81)* | Adherence concerns  (n = 62) | No drug selection concerns  (n = 112)** | At least one drug selection concern  (n = 31) | No adverse drug reaction concerns  (n = 112)** | At least one adverse drug reaction concern  (n = 31) |
| --- | --- | --- | --- | --- | --- | --- |
| Age at assessment, years | 82.8 ± 11.2  (48.7–103.0) | 81.3 ± 10.6 (51.5–96.0) | 82.3 ± 11.2 (48.7–103.0) | 81.7 ± 9.8  (52.8–100.6) | 82.0 ± 11.3 (48.7–103.0) | 82.8 ± 9.2  (60.6–100.6) |
| Age ≥80 years | 55 (67.9%) | 43 (69.4%) | 78 (69.6%) | 20 (64.5%) | 75 (67%) | 23 (74.2%) |
| Charlson Comorbidity Index score | 4.6 ± 2.8  (0–14) | 4.6 ± 2.9  (0–13) | 4.4 ± 2.9  (0–14) | 5.3 ± 2.6  (0–10) | 4.5 ± 2.8  (0–14) | 4.8 ± 3.0  (0–13) |
| Total number of medications upon discharge | 11.7 ± 4.5  (4–25) | 12.2 ± 4.6  (3–25) | 11.7 ± 4.4  (3–25) | 12.6 ± 5.0  (6–25) | **11.4 ± 4.2**  **(3–24)** | **13.8 ± 5.3**  **(6 –25)** |
| Tablet burden upon discharge (excluding ‘when required’ medications) | 13.3 ± 7.0  (4–35) | 14.3 ± 6.8  (2–35) | 13.4 ± 6.7  (2–35) | 14.7 ± 7.6  (2–35) | 13.4 ± 6.9  (2–35) | 15.0 ± 6.9  (4–30) |
| Number of changes to medication upon discharge | 5.6 ± 4.1  (0–17) | 6.6 ± 5.1  (0–25) | 6.0 ± 4.4  (0–20) | 6.1 ± 5.1  (0–25) | 6.2 ± 4.6  (0–25) | 5.6 ± 4.4  (0–16) |
| Using a Webster pack | 46 (56.8%) | 34 (54.8%) | 65 (58.0%) | 15 (48.4%) | 64 (57.1%) | 16 (51.6%) |
| Discharged on an anti-coagulant | 37 (46.3%) | 24 (38.7%) | 49 (44.1%) | 12 (38.7%) | 45 (40.5%) | 16 (51.6%) |
| Discharged on insulin | 7 (8.8%) | 8 (12.9%) | 14 (12.6%) | 1 (3.2%) | 12 (10.8%) | 3 (9.7%) |
| Discharged on anti-cholinergic medication | 8 (10.0%) | 7 (11.3%) | **7 (6.3%)** | **8 (25.8%)** | 10 (9.0%) | 5 (16.1%) |
| Discharged on opioids | 15 (18.8%) | 17 (27.4%) | 25 (22.5%) | 7 (22.6%) | 21 (18.9%) | 11 (35.5%) |
| Discharged on benzodiazepines | 8 (10.0%) | 9 (14.5%) | 11 (9.9%) | 6 (19.4%) | 11 (9.9%) | 6 (19.4%) |
| Number of days before HIMR1 | 7.3 ± 5.4  (0–27) | 7.6 ± 5.8  (1–26) | 7.3 ± 5.2  (1–26) | 8.0 ± 7.0  (0–27) | 7.3 ± 5.5  (0–27) | 7.9 ± 6.1  (1–26) |

^Data are presented as the mean ± SD (range) or n (%)^

^* n = 80, **n = 111 for total number of medications, tablet burden upon discharge, and medication type^

^Significant between-group differences are presented in bold: Discharged on anti-cholinergic medication and drug selection concern identified (^*^p^* ^= 0.002). Increase in the total number of medications upon discharge, and adverse drug reaction concern identified (^*^p^* ^= 0.010)^

**Supplementary Table 2: Patient factors and moderate/high importance of pharmacist intervention at HIMR1**

|  | _No documented actions or actions of mild significance_  _(n = 132)_ | _Moderate/High importance interventions_  _(n = 11)_ |
| --- | --- | --- |
| Age at assessment, years | 82.0 ± 11.3 (48.7–103.0) | 84.8 ± 4.0 (80.4–92.0) |
| Age ≥80 years | **87 (65.9%)** | **11 (100%)** |
| Charlson Comorbidity Index score (CCI) | **4.4 ± 2.8 (0–14)** | **6.6 ± 1.9 (4–10)** |
| Total number of medications upon discharge | 12.0 ± 4.7 (3–25) | 10.6 ± 2.5 (8–15) |
| Tablet burden on discharge (excluding ‘when required’ medications) | 13.8 ± 7.0 (2–35) | 13.2 ± 6.0 (4–28) |
| Number of changes to medication on discharge | 6.1 ± 4.7 (0–25) | 5.6 ± 3.0 (2–13) |
| Using a Webster pack | 75 (56.8%) | 5 (45.5%) |
| Discharged on an anti-coagulant | 57 (43.5%) | 4 (36.4%) |
| Discharged on insulin | 15 (11.5%) | 0(0%) |
| Discharged on anti-cholinergic medication | 13 (9.9%) | 2 (18.2%) |
| Discharged on opioids | 31 (23.7%) | 1 (9.1%) |
| Discharged on benzodiazepines | 15 (11.5%) | 2 (18.2%) |
| Number of days before HIMR1 | **7.6 ± 5.6 (1–27)** | **5.1 ± 4.7 (0–14)** |

^Significant between-group differences are in bold: Age ≥80 years and presence of moderate/high importance interventions (^*^p^* ^= 0.019)^

^Increase in CCI and presence of moderate/high importance interventions (^*^p^* ^= 0.016)^

^Fewer days before HIMR1 and presence of moderate/high importance interventions (^*^p^* ^= 0.049)^
